# Supplementary material for: Heat-Killed Lactococcus lactis subsp. cremoris H61 Altered the Iron Status of Young Women: A Randomized, Double-Blinded, Placebo-Controlled, Parallel-Group Comparative Study
Source: Nutrients. 2022 Jul 30;14(15):3144. doi: 10.3390/nu14153144 (PMC9370818; doi:10.3390/nu14153144)
Supplement: Supplementary file 1 [file nutrients-14-03144-s001.zip › nutrients-1823053-supplementary.pdf]

Supplementary Materials

Table S1. Changes in the iron status of women analyzed by generalized estimating equation.

		Group	Pre					Post					*p*	
			EMM	SE		*p*		EMM	SE		*p*		(Pre vs Post)	
Red blood cell	10^4 cell/mL	H61	464.2	2.4	]	0.455		454.4	8.1	]	0.661		0.239	
		Control	459.2	6.1				460.6	6.7				0.840	
Hemoglobin	g/dL	H61	13.7	0.1	]	0.327		13.3	0.3	]	0.736		0.133	
		Control	13.5	0.2				13.4	0.2				0.751	
Hematcrit	%	H61	42.5	0.2	]	0.404		41.1	0.7	]	0.658		0.057	
		Control	42.0	0.5				41.7	0.6				0.561	
MCV	fL	H61	91.7	0.3	]	0.982		90.5	0.5	]	0.927		0.023	*
		Control	91.7	0.5				90.6	0.5				0.012	*
MCH	fL	H61	29.6 29.5	0.0 0.1	]	0.499		29.3 29.3	0.1	]	0.894		0.024	**
		Control							0.1				< 0.001	
MCHC	%	H61	32.2	0.1	]	0.883		32.3	0.2	]	0.832		0.410	
		Control	32.2	0.2				32.3	0.2				0.654	
Serum iron	μg/dL	H61	103.8	4.4	]	0.025	*	140.8	17.1	]	0.022	*	0.035	*
		Control	75.2	11.8				72.8	13.7				0.835	
TIBC	μg/dL	H61	358.9	4.5	]	0.424		346.7	9.0	]	0.528		0.140	
		Control	368.6	8.7				357.5	9.0				0.089	
UIBC	μg/dL	H61	256.8	4.0	]	0.001	**	221.3	10.8	]	0.003	**	< 0.001	**
		Control	288.3	8.3				278.6	9.6				0.367	
TSAT	%	H61	28.8	1.0	]	0.008	**	35.5	3.4	]	0.028	*	0.049	*
		Control	21.9	2.4				22.1	3.0				0.947	
Ferritin	ng/mL	H61	31.1	0.9	]	0.209		35.9	1.3	]	0.018	*	< 0.001	**
		Control	28.3	1.5				29.4	1.5				0.407	
Hepcidin	ng/mL	H61	7.8	0.7	]	0.314		7.2	1.4	]	0.300		0.659	**
		Control	5.9	1.3				4.5	1.3				0.003	

EMM, estimated marginal mean; SE, standard error; TIBC, total iron-binding capacity; UIBC, unsaturated iron binding capacity; TSAT, transferrin saturation; MCV, mean corpuscular volume; MCH, mean corpuscular hemoglobin; MCHC, mean corpuscular hemoglobin concentration. * $p < 0.05$; ** $p < 0.01$.

Table S2. Iron status and menstrual cycle analyzed by generalized estimating equation.

		Folicular phase		Luteal phase		p	
		EMM	SE	EMM	SE		
Red blood cell	10^4 cell/mL	454.0	2.7	465.2	3.8	0.084	
Hemoglobin	g/dL	13.3	0.1	13.6	0.1	0.120	
Hematcrit	%	41.3	0.2	42.3	0.4	0.119	
MCV	fL	91.3	0.3	90.9	0.3	0.602	
MCH	fL	29.4	0.1	29.3	0.1	0.465	
MCHC	%	32.2	0.1	32.3	0.1	0.912	
Serum iron	µg/dL	99.2	7.6	102.4	8.6	0.843	
TIBC	µg/dL	348.6	4.7	367.2	7.3	0.119	
UIBC	µg/dL	253.3	4.3	269.2	4.7	0.074	
TSAT	%	27.4	0.0	26.8	0.0	0.807	
Ferritin	ng/mL	33.9	1.0	28.5	1.2	0.015	*
Hepcidin	ng/mL	7.9	0.7	4.8	1.1	0.085	

EMM, estimated marginal mean; SE, standard error; TIBC, total iron-binding capacity; UIBC, unsaturated iron-binding capacity; TSAT, transferrin saturation; MCV, mean corpuscular volume; MCH, mean corpuscular hemoglobin; MCHC, mean corpuscular hemoglobin concentration. * $p < 0.05$

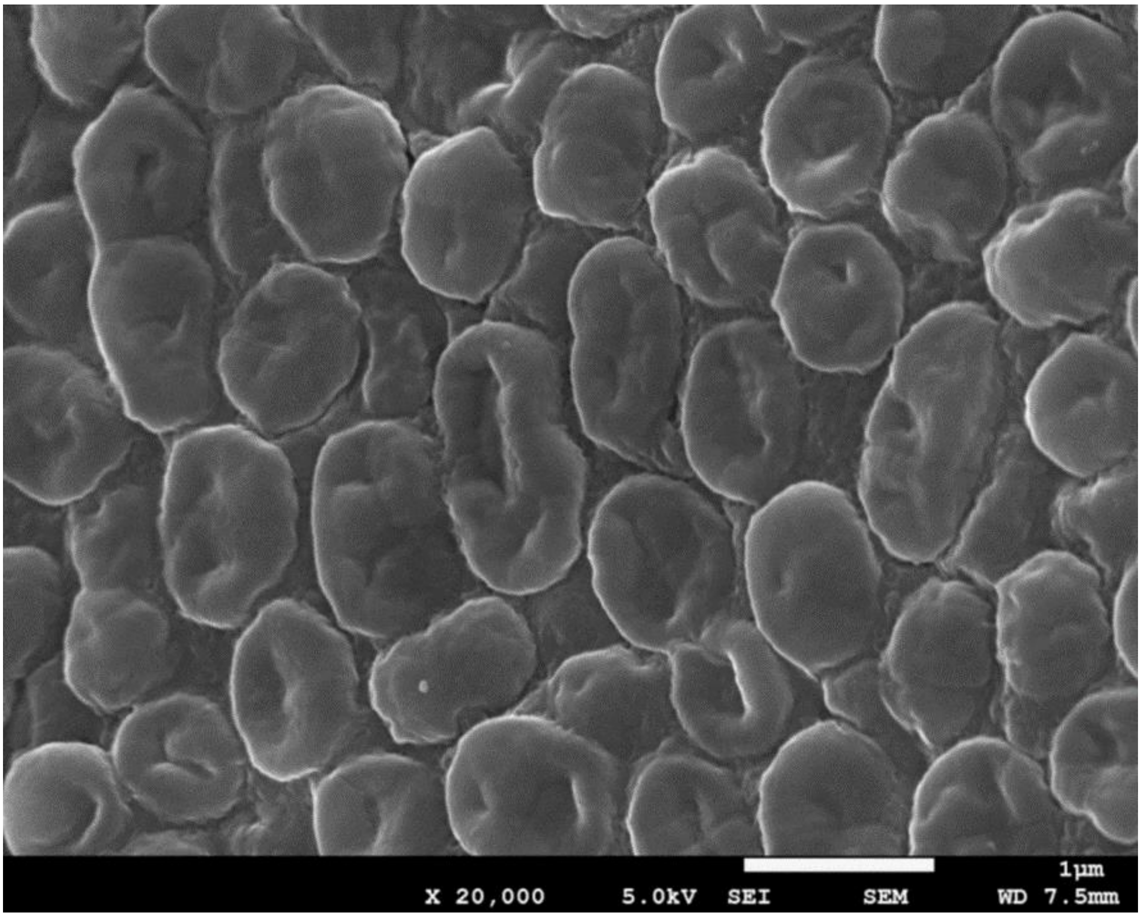

Figure S1. Microscopic view of the heat-killed *Lactococcus lactis* subsp. *cremoris* H61.
